# Chaotic Signatures Exhibited by Plasmonic Effects in Au Nanoparticles with Cells

**DOI:** 10.3390/s19214728

**Published:** 2019-10-31

**Authors:** Hilario Martines-Arano, Blanca Estela García-Pérez, Mónica Araceli Vidales-Hurtado, Martín Trejo-Valdez, Luis Héctor Hernández-Gómez, Carlos Torres-Torres

**Affiliations:** 1 Sección de Estudios de Posgrado e Investigación, Escuela Superior de Ingeniería Mecánica y Eléctrica Unidad Zacatenco, Instituto Politécnico Nacional, Ciudad de México 07738, Mexico; hilario.martines.arano@gmail.com (H.M.-A.); luishectorhdzgom56@gmail.com (L.H.H.-G.); 2 Departamento de Microbiología, Escuela Nacional de Ciencias Biológicas, Instituto Politécnico Nacional, Ciudad de México 11340, Mexico; blanch.gp18@gmail.com; 3 Centro de Investigación en Ciencia Aplicada y Tecnología Avanzada Unidad Querétaro, Instituto Politécnico Nacional, Santiago de Querétaro, Querétaro 76090, Mexico; mvidales@ipn.mx; 4 Escuela Superior de Ingeniería Química e Industrias Extractivas, Instituto Politécnico Nacional, Ciudad de México 07738, Mexico; martin.trejo@laposte.net

**Keywords:** plasmonics, nonlinearity, Rössler systems, spectroscopy, electrical sensing, thin films

## Abstract

The evolution of the optical absorptive effects exhibited by plasmonic nanoparticles was systematically analyzed by electronic signals modulated by a Rössler attractor system. A sol-gel approach was employed for the preparation of the studied Au nanoparticles embedded in a TiO_2_ thin solid film. The inclusion of the nanoparticles in an inhomogeneous biological sample integrated by human cells deposited in an ITO glass substrate was evaluated with a high level of sensitivity using an opto-electronic chaotic circuit. The optical response of the nanoparticles was determined using nanosecond laser pulses in order to guarantee the sensing performance of the system. It was shown that high-intensity irradiances at a wavelength of 532 nm could promote a change in the absorption band of the localized surface plasmon resonance associated with an increase in the nanoparticle density of the film. Moreover, it was revealed that interferometrically-controlled energy transfer mechanisms can be useful for thermo-plasmonic functions and sharp selective optical damage induced by the vectorial nature of light. Immediate applications of two-wave mixing techniques, together with chaotic effects, can be contemplated in the development of nanostructured sensors and laser-induced controlled explosions, with potential applications for biomedical photo-thermal processes.

## 1. Introduction

Owing to their simplicity and the advantages exhibited by chaotic functions, Rössler systems have been used in many technological applications [1]. These approaches are intended to improve the specific properties of dynamic processes related to stability conditions and controlled chaos [2]. Chaotic characteristics monitored for instrumentation are useful because they present a considerable increase in the sensitivity of measurements. Different tools for observing the data involved in chaos theory have been employed in synchronization technology [3]. The chaos in nonlinear electronic circuits has been a topic of interest, owing to its unique features which are derived from the initial conditions for measurement. In particular, qualitative studies for stochastic oscillators [4], encryption [5], fractal sampling [6], wireless sensors [7], molecular medicine [8], and neural networks [9] based on chaos have been reported.

In recent years, numerous researchers have proposed a variety of approaches for developing Lorenz and Rössler systems in order to use them as an elemental configuration [10]. One of the fundamental conceptions of a three-dimensional Rössler system is that it is the simplest model for continuous-time chaos. In this regard, Rössler equations can be considered simplifications of Lorenz attractors [11]; with this in mind, the study of chaotic signals and their modulation for sensing have revealed the emergence of several contrasting themes. While some researchers consider chaotic systems as deterministic tools that generate random and unpredictable time series, others argue that chaotic systems are predictable. However, as a consequence of the dependence on the sensitivity of the initial conditions, chaotic attractors display remarkable advantages for testing unknown signals. Examples of suitable chaotic systems for ultrafast signal processing [12], remote sensing images [13], transformation of biological DNA sequences [14], microwave sensing [15], all-optical codification [16], probabilistic [17], telecom [18], artificial intelligence [19], and quantum functions [20] have been proposed.

In nanoscience, outstanding experiments exhibiting powerful nonlinear optical responses arising from chaotic behavior have been carried out in the areas of photonics [21] and plasmonics [22]. Some interesting observations been made, e.g., that chaotic Rössler systems strongly increase the level of sensitivity of complex measurements in nanostructured thin films [23]. Some of these results may be related to the magnitude of irradiation [24]. It is worth mentioning that nanostructures have a strong presence in many technologies, given their relevance in the integrated circuit industry. Metal nanoparticles (NPs) can manipulate optical irradiation on an ultrashort time scale [25]; an essential aspect for this characteristic is their localized surface plasmon resonance (LSPR) that exerts a strong influence on quantum and optical interactions [26]. Plasmonic effects give rise to the possibility of transforming optical irradiation into thermal energy by fundamental low-dimensional interactions [27]. A strong impact in tunable thermal focusing can be derived by nanoscale thermo-plasmonics [28], and potential applications in phototherapy, imaging, drug delivery, surgery, sensing, or communication tools, among others, can be envisioned.

The incorporation of Au NPs in biological samples as part of the design process of non-destructive optical techniques has been the object of increasing popularity in recent times [29,30,31,32]. In light of recent research related to Au NPs and chaos theory, it seems suitable to analyze these findings in some important areas of science and technology.

In this direction, in this work, an attempt has been made to further explain the optical, photo-thermal, and electrical characteristics exhibited by plasmonic NPs interacting with chaotic signals. Thin solid film samples integrated by TiO_2_-supported Au NPs were studied. An electronic modulator governed by Rössler equations was implemented to identify the electronic effects in the sample irradiated by light. The inclusion of NPs into human cells deposited onto a highly conductive ITO matrix was explored using our chaotic technique. We believe that these findings may be a basis for designing plasmonic and opto-electronic devices which will be improved by chaos theory.

On the other hand, in order to guarantee the stability of the nanostructures during the sensing experiments, nanosecond pulsed interactions were analyzed to describe the photothermal effects and potential modification of the samples by high-intensity optical irradiations. We demonstrated that under high-intensity conditions of laser irradiance, our laser system was able to promote an increase in the Au NP density in a TiO_2_ thin film. Finally, vectorial two-wave mixing experiments were carried out to analyze the evolution of the ablation threshold of biological samples. Potential applications for the instrumentation of multi-scale signals are proposed.

## 2. Materials and Methods

### 2.1. Synthesis of the Au NPs in a TiO_2_ Film

Titanium dioxide solid films with Au NPs were prepared by a sol-gel method [33]. The Au NPs were synthesized in situ through direct radiation in the laboratory. Initially, the TiO_2_ solution was prepared using 0.03 mol of precursor. The precursor solution was firstly dissolved in 200 mL of absolute ethanol, and then mixed with a 30% *v*/*v* of water-ethanol. Once the mixture was prepared, it was necessary to adjust its pH to 1.25 using hydrochloric acid. The resulting mixture was incorporated into a standard solution of an Au precursor with a volume of 0.7 mL using a metal concentration of 1000 mg/L. An ultraviolet lamp source was used to promote the formation of NPs in the solution. Film samples with an average thickness of 200 nm were prepared by a spin coating technique. Spectrophotometric measurements were undertaken with a Perkin Elmer UV/VIS XLS system. High-Resolution Transmission Electron Microscopy (HRTEM) analysis was carried out with a JEM—ARM200CF&Gatan Ultrascan 1000XP system. The electrical impedance of the samples was measured by an Autolab PGSTAT302N station; this device is a high power potentionstat/galvanostat. The impedance spectra were measured with a 10 mV signal on a 5 mm^2^ surface of the studied samples and with an integration time of 1 s.

### 2.2. Preparation of the Biological Sample

A human osteoblasts cell line (MG-63 ATCC^®^ CRL-1427™) derived from osteosarcoma was obtained from the American Type Culture Collection (ATCC, Rockville, Maryland, USA). To evidence the actin cytoskeleton of the osteoblasts, monolayers were prepared on a glass coverslip. Osteoblasts monolayers were fixed with 4% paraformaldehyde for 1 h at room temperature. Then, cells were washed twice with Hank’s balanced saline solution (HBSS) and covered with 80 ng of a rhodamine-phalloidin (Sigma-Aldrich, St. Louis, MO, USA). Excess phalloidin was removed by washing 3 times with HBSS. Finally, the labeled cells were mounted on a glass slide with Vectashield-DAPI (4’, 6-diamidino- 2-phenylindole) (Vector Laboratories, Inc., Burlingame, CA, USA) and observed with a confocal system coupled to an inverted microscope (LSM5 PASCAL Zeiss, Jena, Germany).

### 2.3. Modeling of Chaotic Electronic Rössler System

The sensitivity of the film with respect to the optical irradiation was indirectly explored by monitoring electronic signals through the sample. Chaotic signals are capable of instantaneously testing the electromagnetic conditions of a sample that are dependent on the electrical frequency of the signals. The optical source was provided by the second harmonic of a Nd:YAG laser system (Continuum Model SL II) at 532 nm wavelength, 4 ns pulse duration, 10 mJ of pulse energy, and 1 mm of focused beam waist in a single-beam configuration of irradiation. To implement the electronic modulation system, we employed the Rössler differential equations that describe a continuous-time dynamics with chaotic behavior [34]:

(1) $$\frac{dx}{dt}=-\left(y+z\right)$$

(2) $$\frac{dy}{dt}=\left(x+ay\right)$$

(3) $$\frac{dz}{dt}=b+z\left(x-c\right)$$

where *a*, *b*, *c* ∈ℝ and they are considered positives and dimensionless values, while *x*, *y*, and *z* denote the state variables of the system. 

**Figure 1:**
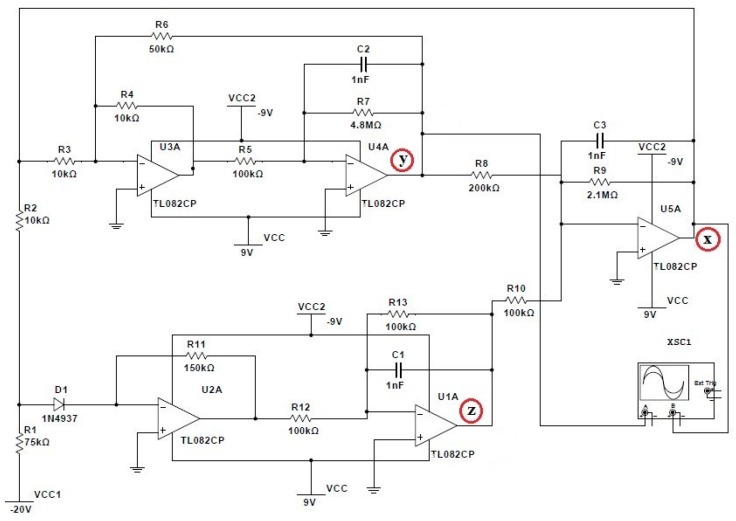
Schematic circuit for the implementation of a Rössler attractor system.

An electronic circuit which was determined according to Equations (1)–(3) was implemented as shown in Figure 1 [35]. We modeled the behavior of the chaotic dynamical systems with the purpose of observing the variations in the attractor according the manipulation of electrical parameters. The circuit shown in Figure 1 is based on the well-known chaotic Rössler equations. In Figure 1, *VCC*, *VCC1*, and *VCC2* refer to the direct current voltage used on the chaotic electrical circuit, i.e., *VCC* = 9 V, *VCC1* = −20 V, and *VCC2* = −9 V. TL082CP is an input operational amplifier JFET in 8 pin DIP packages. This device incorporates high voltage JFET and bipolar transistors in a monolithic integrated circuit; it is represented by U1A-U5A. XSC1 represents a digital oscilloscope. *D*1 is a 1N4937 diode; it is used for fast switching rectification of the power supply in this electronic circuit. The symbol *R* expresses the resistance of the electronic circuit; resistances values *R*1–*R*13 vary from 10 to 4.8 kΩ. *C*1–*C*3 correspond to capacitors, and all of which are 1 nF. 

In Figure 1, variables *x*, *y*, and *z* are indicated by a red circle. The *x* point in the circuit corresponds to the output of the operational amplifier U5A, whereas the *y* point is indicated by the output of operational amplifier U4A. The *z* point is represented by the output of the operational amplifier U1A. The Rössler system values for *x*, *y*, and *z* are dynamic variables which evolve over time. The parameters *x*, *y*, and *z* determine the phase space; therefore, these values change according the system behavior [36]. The chaotic Rössler system in this work was conceived considering *a* = 0.35, *b* = 10, and *c* = 24 in Equations (1)–(3) as the initial conditions. The chaotic Rössler system was designed with three fixed points at:

(4) $$x=\frac{1}{2}\left(\frac{c}{a}\pm \frac{\sqrt{c^{2}-4ab}}{a}\right)$$

(5) $$y=-\frac{1}{2}\left(\frac{c}{a}\pm \frac{\sqrt{c^{2}-4ab}}{a}\right)$$

(6) $$z=\frac{1}{2}\left(\frac{c}{a}\pm \frac{\sqrt{c^{2}-4ab}}{a}\right)$$

### 2.4. Modification of the LSPR of the Sample by Nanosecond Pulses

**Figure 2:**
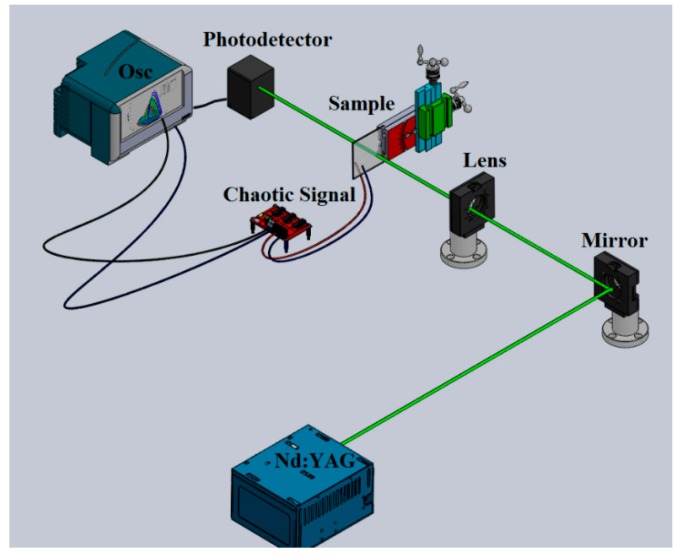
Schematic illustration of the nanosecond single-beam irradiance experiment.

The 532 nm wavelength of our nanosecond Nd:YAG laser system with a beam waist of 6 mm was employed in a single-shot mode. Figure 2 schematizes the experimental setup where a dielectric mirror, an oscilloscope, a focal lens, and the PIN photodetector are identified in the illustration. An electronic modulator connected to the sample described in Section 2.2 is depicted in the picture as a chaotic signal circuit used to explore the photoinduced changes of the sample under irradiation. The maximum pulse energy employed at the laser output in the experiment was 68 mJ. 

### 2.5. Biosensing Assisted by Au NPs and Steady-State Rössler Attractors

The principle underlying this function is schematically illustrated in Figure 2. We used the experimental setup presented in Figure 2 in order to evaluate the presence of Au NPs in a biological sample represented by human osteoblast cells deposited onto a quartz substrate. The irradiance of the Nd:YAG laser system was limited to avoid optical damage in the cells and any structural or morphological modification in the Au NPs. The calibration of the steady-state Rössler attractors emerging from the electronic system was carried out by considering the propagation of a beam through biomarked cells with Au NPs in living conditions. The unsteady-state of the Rössler system potentially describes the decay of the cells or the absence of the Au NPs in the samples.

### 2.6. Laser Ablation Threshold in the Biomarkers under a Vectorial Two-Wave Mixing Irradiation

In order to analyze the modification in the ablation threshold of the Au NPs incorporated into the cells, a two-wave mixing experiment was conducted. The optical damage in the sample was regulated by the modification of the contrast in the irradiance fringe pattern obtained by the superposition of two coherent pulses provided by our nanosecond Nd:YAG laser. The angle between the linear polarizations of the incident beams impinging into the sample was controlled by a half-wave plate [37]. The geometrical angle of the incident beams was close to 20°, with a maximum total irradiance of 100 mJ. We used focused beams of approximately 1 mm diameter in a single-shot mode. The study of the distribution that corresponds to the electric field in the excited NPs located in the bright fringes of the interaction was computed considering the Fresnel-Kirchhoff diffraction integral [38],

(7) $$U\left(\eta ,\varsigma ,\zeta \right)=\frac{1}{i\lambda }\overset{}{\underset{S}{\iint }}U\left(\eta_{0},\varsigma_{0}\right)\left[\frac{cos\left(\vec{n},r\right)-cos\left(\vec{n},r^{\prime }\right)}{2}\right]\frac{e^{i\kappa r}}{r}ds$$

where λ is the wavelength, *U* (*η*_0_, *ς*_0_) is the mathematical description of the amplitude distribution located at the ζ = 0 position *S*, with a normal direction $\vec{n}\;$, *r* and *r*’ are the vector located between point ζ = 0 and a normalized point in the plane ζ, and κ = 2π/λ is the wavevector employed in the numerical simulations.

## 3. Results

**Figure 3:**
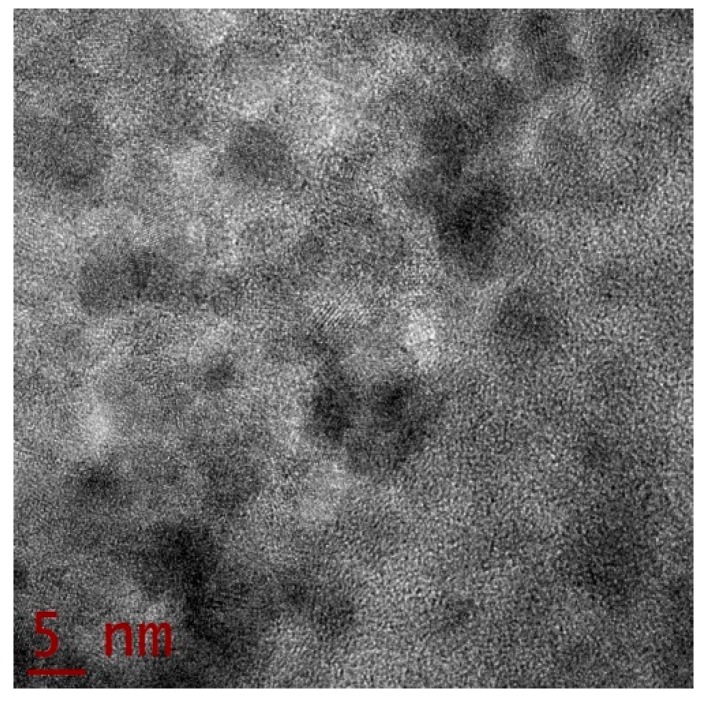
Representative HRTEM micrograph of a region of the studied thin solid film sample showing the Au NPs in dark color.

Figure 3 shows a panoramic HRTEM image of the studied thin film in bright field mode. Dark points represent segregated Au NPs in the micrograph. According to HRTEM studies, clear evidence of a no-nucleation regime of the Au NPs on the solid thin film surface can be guaranteed. From statistical analyses of the HRTEM images, it is possible to conclude that the film has an inhomogeneous morphology with randomly-distributed NPs. The Au NPs have a quasi-spherical shape. According to our statistical measurements, the size of the studied NPs has an approximate range of between 4 and 12 nm.

**Figure 4:**
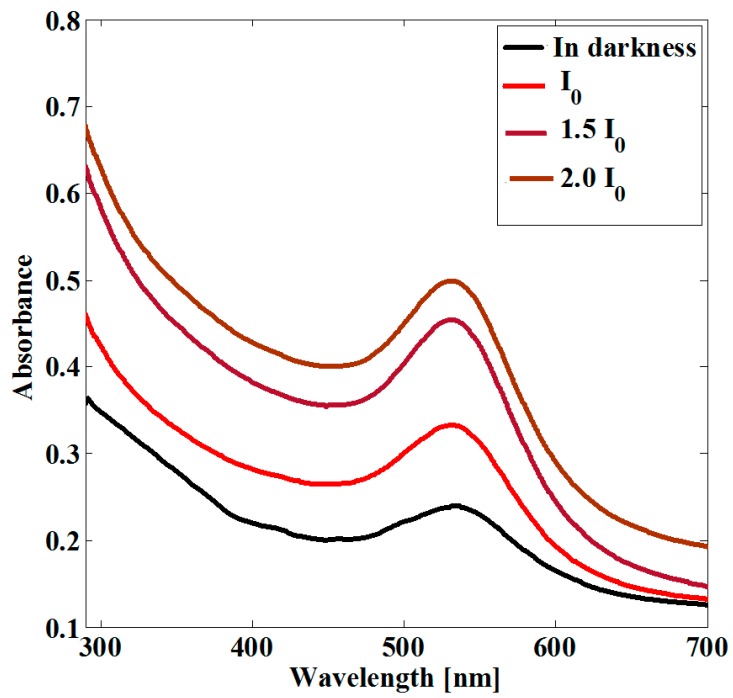
UV-vis absorbance spectra of the studied sample before and after nanosecond pulsed irradiation at 532 nm.

In Figure 4, the absorbance spectra of the studied sample, before and after nanosecond pulsed irradiation, is represented. The absorption band related to the LSPR of the Au NPs is close to the 532 nm excitation wavelength provided by the laser system. The incident pulsed laser energy employed corresponded to *I*_0_ = 2.1 GW/cm^2^. Figure 4 reveals a gradual irreversible increase in the LSPR absorbance as a function of the optical irradiation.

It should be noted that the spectral shift that can be observed in Figure 4 is directed towards the blue part of the spectrum. This shift is assumed to be due from the formation of smaller NPs on the sample. The possibility of increasing the nanoparticle density in particular samples due to the heat caused by laser irradiation [39] has been previously reported. The release of metal atoms for the creation of additional nanoparticles in nanocomposites may occur due to heat; and due to an adsorption process, the average size of the NPs automatically changes [40]. The modification in the absorption band associated with the LSPR in nanocomposites irradiated by a laser system can be considered as a result of a decrease in the mean size and change in density of the NPs that become integrated into the sample [41].

A nonlinear change in the LSPR absorption band results from the energy deposited by laser irradiation. The energies employed for irradiation correspond to the photothermally-activated process to promote the formation of metallic NPs by thermal annealing [42]. Due to the heat caused by laser irradiation and the heightening of the absorbance band of the LSPR, it is expected that an adsorption process may be responsible for the increase in the nanoparticle density of the samples. 

Furthermore, research into the irradiation of thin films by different pulse durations has a long history, showing changes in the optical absorbance spectra of the samples [43]. Our experimental results match previous measurements in comparative samples [44,45].

The absorption band exhibited by the LSPR of monometallic NPs changes, with a strong dependence on size and shape [46,47,48]. It is important to mention that this fascinating characteristic can be useful in the design of particular applications for instrumentation and sensors functions.

**Figure 5:**
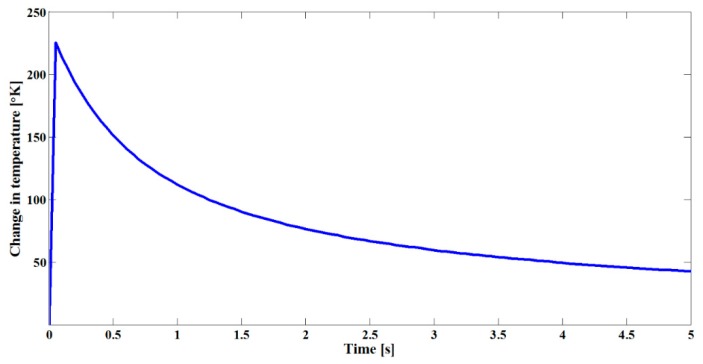
Numerical curve for temperature changes exhibited by Au NPs under optical irradiation vs. time.

Numerical calculations related to heat diffusion were carried out in order to estimate the increase in temperature during the laser irradiation of the sample. The basis for these calculations is the heat conduction equation [49].

(8) $$\frac{\partial T}{\partial t}=\frac{\partial }{\partial \zeta }\left[\frac{k}{\rho C}\frac{\partial T}{\partial \zeta }\right]+\frac{\alpha }{\rho C}I\left(t,\zeta \right)$$

where *T* is temperature as a function of the propagation length *ζ*, and *I* represents the optical irradiance; we used *ζ* = 180 nm, the thermal conductivity *k* = 250 W·m^−1^·K^−1^, the density *ρ* = 1 × 10^−3^ kg/cm^3^ and the heat capacity *C* = 1 × 10^3^ J·Kg^−^1·K^−1^. Furthermore, the time irradiation exposure for the sample was defined as *t* = 5 s, whereas the linear absorption coefficient calculated by considering the LSPR spectra was α = 3 × 10^6^ m^−1^. Figure 5 shows a numerical simulation related to the heat transference in our sample irradiated by nanosecond pulses, as described by Equation (8). According to the numerical results, the temperature increases at the region where the Au NPs were located, evidencing laser absorption; this confirms the increase in temperature in this process featuring about 220 K.

**Figure 6:**
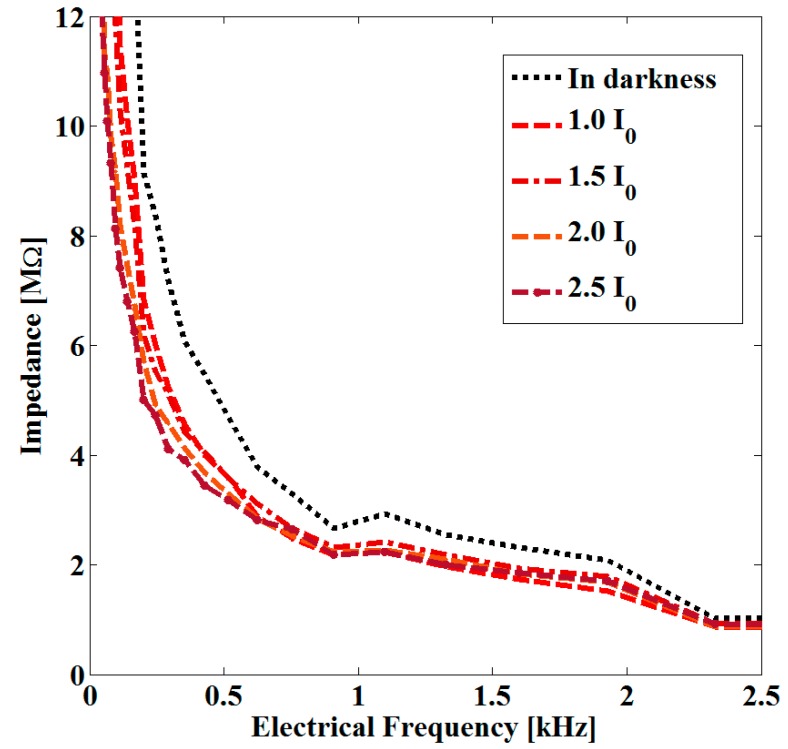
Impedance spectra of the studied sample before and after nanosecond pulsed irradiation at 532 nm.

Regarding the modification in the optical properties of the sample, we consider that electrical properties can also be modified by the optical irradiation employed. The increase in the density of Au NPs should increase the conductivity of the sample and decrease its electrical impedance. In good agreement with this, Figure 6 illustrates the variation in the magnitude of the electrical impedance as a function of electrical frequency for the irradiated sample. A decrease in the magnitude of the electrical impedance of the sample with the increase of the electrical frequency can be seen in Figure 6; this behavior could be modeled in the simplest form as a resistor and a capacitor in a series circuit.

**Figure 7:**
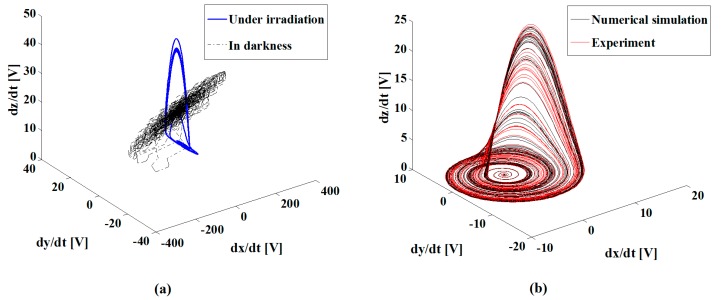
Experimental and numerical data obtained by chaotic modulation in conductivity measurements. (**a**) Comparison of one experimental measurement on the edge stability of the system, and another with unstable behavior; the first one under irradiation and the second one in darkness; (**b**) Steady-state Rössler attractors obtained in the studied sample.

Since the optical and electrical properties of the sample were simultaneously modified by laser irradiation, we expected a potential photoconductivity in the sample that could be present due to the mobility of the Au NPs under low-irradiance illumination. The high sensitivity of a measuring system with the advantages of electronic chaotic modulation was then proposed. We explored the conductivity of the film using different carbon electrodes; the scheme is presented in Figure 2. The experimental results shown in Figure 7a clearly describe the evolution of the Rössler attractor behavior integrated by electronic signals exposed to particular irradiation conditions in the sample. The steady-state of the system is illustrated in Figure 7a; it was assigned the value of *I*_0_/100 in the TiO_2_ film without NPs. The conditions for the stability of the system are calibrated according to a reference that may represent the electrical and optical characteristics of a particular region of the sample. The instability of the system is also depicted in Figure 7a; this corresponds to the sample measured in darkness. The incorporation of Au NPs into the TiO_2_ film generates a well-defined orbit that describes stability, as shown in Figure 7b. Moreover, photoconductivity was confirmed by the steady-state of the chaotic attractor controlled by the increase of the irradiance in the Au NPs doping the TiO_2_ sample.

**Figure 8:**
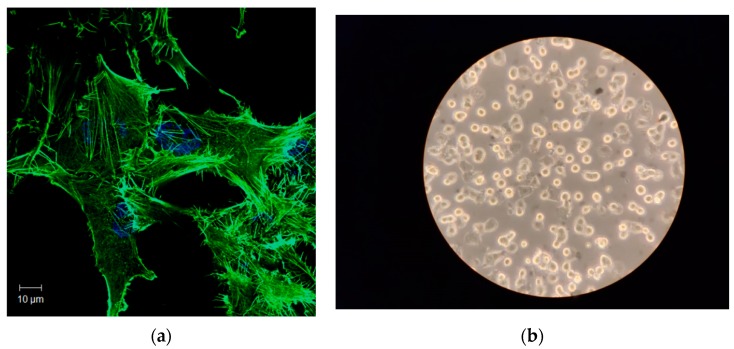
Representative images of the studied human osteoblasts. (**a**) Confocal micrograph. Actin cytoskeleton was labeled with rhodamine-phalloidin (green) and cells nuclei were stained with DAPI (blue); (**b**) Microscopic image of the studied human osteoblasts deposited in the substrate.

To further investigate the potential of the proposed technique for the detection of plasmonic effects, the Au NPs were incorporated into a biological sample comprising human osteoblasts cells. Figure 8a illustrates a representative confocal image of human osteoblasts; Figure 8b shows cells deposited in an ITO substrate from a conventional optical microscope.

Figure 8 shows that these cells exhibit long actin filaments and short actin filaments resembling filopodia structures. Filopodia-like structures can be observed as membrane protrusions that extend out from the cell edge.

**Figure 9:**
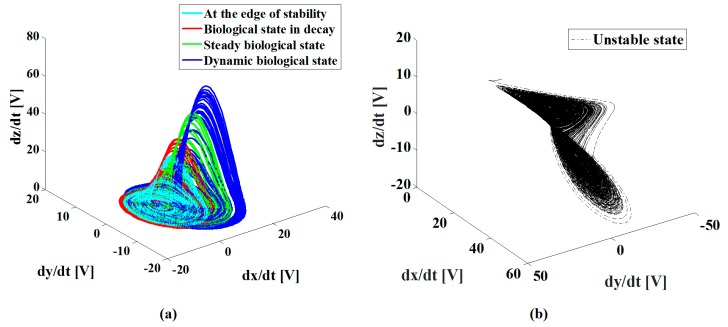
(**a**) Evolution of the steady-state Rössler attractors obtained in the Au NPs incorporated into cells; (**b**) Measurement obtained in dead cells.

In order to further describe the importance of this work, we used the proposed technique to identify the biological conditions exhibited by the human osteoblasts. The cells were deposited into an ITO substrate, and Au NPs were incorporated into specific cells by a functionalization process. Figure 9 shows the chaotic attractors that correspond to cells in constant motion (dynamic biological state), in a steady biological state, in an irreversible decay of their biological state, and in the limit of their biological conditions of life. We ensured that the NPs did not change during the sensing experiments. The dynamics of living cells in the measurements give evidence for the evolution of the chaotic attractors in Figure 9. Figure 9b shows the unstable data measured by the chaotic system when it was monitoring the deaths of the studied cells. When the biomarked cells were isolated from feeding and living conditions, i.e., pressure and temperature, their optical absorption strongly changed; this situation was immediately identified by our system.

Figure 9 shows the remarkable changes exhibited by the attractor system’s behavior when an evolution of its biological conditions took place in the measurement of the sample. The potential of this technique for monitoring biological mechanisms and for characterizing synergistic processes can be thereby envisioned.

In this work, we are sensing changes in conductivity which are induced by the absorption of light in the samples. We use a chaotic electric effect as an indirect sensor of the optical absorption properties of the studied samples. As the NPs are illuminated by our system, the LSPR are excited, and the opto-electrical response of the NPs changes; then, a change in the attractor is observed. However, for an excitation wavelength of 532 nm, there was also a reduction of approximately 0.1 OD for the absorbance exhibited by dead cells in comparison to live cells; this condition also changed the electrical response of the system to be detected, as illustrated in Figure 9.

**Figure 10:**
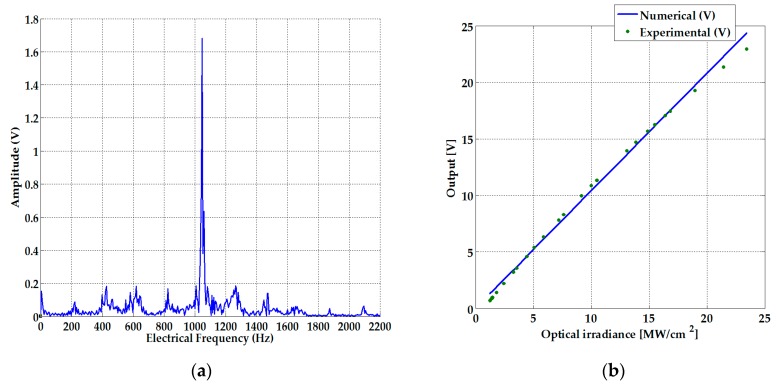
(**a**) Fourier spectrum of the steady-state Rössler attractor signal; (**b**) Calibration curve for the sensitivity calculation.

Since the Au NPs samples are dependent on electrical frequency, we plotted in Figure 10b the Fourier spectrum of the steady-state chaotic signal employed in order to evaluate photo-induced properties. From Figure 10a, it can be clearly deduced that a chaotic modulation can improve the sensing capabilities of the system by testing a group of electrical frequencies in comparison to the response that may be associated with an electrical sensor working with a continuous wave signal in order to carry out measurements. To numerically determine the sensitivity of the chaotic system, the calibration curve was evaluated. The sensitivity for our chaotic system was ±1.04 V/(MW/cm^2^), as illustrated in Figure 10b.

The calibration curve shows the experimental data far from the potential photothermal damage to the samples. We used pulses of 4 nanoseconds, which provide laser fluences below 100 mJ/cm^2^.

To further investigate the identification of particular plasmonic conditions of biomarkers incorporated into biological samples, we explored the vectorial nature of light in Au NPs with isolated cells with a two-wave mixing experiment. It should be noted that the ablation threshold can be dependent on the size and distribution of the NPs, since the LSPR excitation strongly depends on morphology of the nanostructures. In this work, the concentration of the Au NPs incorporated into the cells was heuristically chosen by monitoring the UV-vis spectra in order to better observe the absorption band of the LSPR. Additional measurements related to the electronic chaotic behavior of the samples were conducted in order to better guarantee an equivalent concentration of NPs in the biological samples explored during ablation measurements.

**Figure 11:**
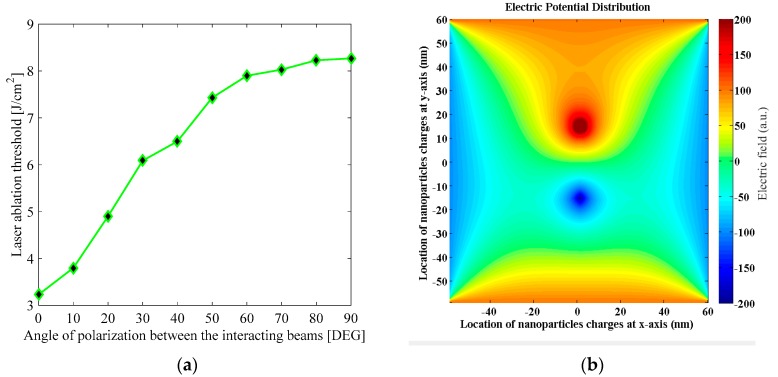
(**a**) Laser ablation threshold as a function on the angle between the planes of polarization of the incident beams in the cells with incorporated Au NPs; (**b**) Electric potential distribution field in a pair of Au NPs.

The laser ablation threshold of the samples was measured in single-shot mode through the superposition of two linearly-polarized nanosecond pulses at 532 nm. The corroboration of the ablation was guaranteed by microscopic observations of the irradiated samples. It was systematically verified that a larger concentration of plasmonic NPs in the cells provided a lower ablation threshold, given that the optical absorbance at a 532 nm wavelength increased according the nanoparticle density. Nevertheless, for the increase of NP concentration in the samples, nonlinear optical effects related to multi-photonic absorption and self-focusing phenomena should be considered too. Finally, Figure 11a shows the modulation of the ablation threshold by rotating the angle of polarization of one of the incident beams in the interaction. The rotation of the polarization gives rise to a variation in the contrast of the irradiance fringes that are generated by the interferometric addition of the electric fields of the beams. For the orthogonal polarizations of the incident beams, 90° in the plot, inhibition of the intensity fringes was obtained, and a homogeneous Gaussian beam irradiated the sample. In contrast, for parallel polarizations, 0° in the plot, bright and dark fringes were generated and a strong reduction of the ablation threshold was reached. According to the modification of the electrical response with irradiance from the results plotted in Figure 6, we can consider that electronic transport must be an important factor in this sample. Electronic and ionic heating in plasmonic NPs may be responsible for the ablation effects under illumination by a nonlinear optical signal. Regarding the advantages of the vectorial nature of interferometry for the promotion of photothermally-controlled actions, a two-wave mixing method assisted by chaotic feedback was proposed to induce sharp selective thermo-plasmonic damage in biomarkers. The observed change in the ablation threshold seems to be responsible for the significant role of the thermo-plasmonic effects that could be expected by the increase in the intensity in the constructive region of the interference of the beams, as has been previously observed for Au NPs under two-wave mixing irradiation [50]. The Mie theory and the Fröhlich condition that state that an excited particle is in resonance when the permittivity of environment surrounding the particle is opposite in sign and twice as large in magnitude as the permittivity of metal. In this regard, if the shape and position of the NPs change, the localization and enhancement of the light at the interface where the particle is located would be changed too. Figure 11b depicts numerical simulations with which to describe the electric field intensity exhibited by the AuNPs, as calculated by Equation (7). As shown in the plot, an intensification in the electromagnetic action can be seen, together with the inhibition of the electromagnetic effect exhibited for a pair of NPs in interaction with each other.

The ablation threshold can be tuned not only by wavelength, pulse duration, and deposited energy, but also by the polarization, which is an intrinsic property of light. The deposited energy in anisotropic samples may be dependent on polarization, and interferometrically-controlled interactions can play a key role in thermal transfers for the photoinduced effects of multi-wave processes. Wavefront shaping by interferometric effects can be also considered for the modification of energy transfer mechanisms based on modulations of the polarization; remarkably, the regulation can be seen of the transfer of angular momentum in the optical manipulation of biological systems by polarization [51]. The possibility of achieving a high degree of control in laser energy transfers is crucial for technological applications where ablation or real-time processes are required. In this work, it was demonstrated that monitoring methods based on chaotic behavior could be useful to provide highly sensitive information associated with the collective oscillations exhibited by plasmonic NPs and interferometric effects. In the progress of instrumentation systems, promises of chaotic systems with which to determine photo-electronic signals have been previously made [52]. Multi-photon interactions, together with chaotic attractor signals, can be also considered in the designs of sensors and actuators devices [53]. The recording of optical effects by chaotic phenomena could be seen as a precursor for the manufacture of optical materials and multi-scale devices [54]. Previous research related to Rössler attractor applications on nonlinear instabilities on nanostructures and chaotic motion has been reported [35], and indications for the development of sensors based on chaotic systems have also appeared in the literature [55]. However, in this study, the system presents the advantage of being predictable, and reaches a notable level stability under well-defined conditions. In this work, it was highlighted that chaotic systems and interferometric signals for reading and writing information in light could offer a basis for future research for the sensing and instrumentation of plasmonic and low-dimensional signals.

## 4. Conclusions

Herein was presented a high-sensitivity method based on an electronically-modulated Rössler system for measuring small changes in plasmonic signals recorded with the assistance of near-resonance optical excitations. The analysis was undertaken by a straightforward comparison of chaotic electronic attractors exploring the sample. The sensitivity of this technique in identifying particular conditions of biological cells was verified by the incorporation of Au NPs in an inhomogeneous sample deposited onto an ITO substrate. We delimited the optical energy for the sensing performance of the proposed system by analyzing the potential evolution of the LSPR associated with monometallic NPs irradiated by laser pulses. We demonstrated that opto-electrical measurements can be employed to analyze particular conditions of biomarked cells with the assistance of electronic chaotic modulation. These findings demonstrate the possibility of designing opto-electronic devices which would be improved by the incorporation of chaos theory.
